# Endogenous Wnt/β-Catenin Signaling Is Required for Cardiac Differentiation in Human Embryonic Stem Cells

**DOI:** 10.1371/journal.pone.0011134

**Published:** 2010-06-15

**Authors:** Sharon L. Paige, Tomoaki Osugi, Olga K. Afanasiev, Lil Pabon, Hans Reinecke, Charles E. Murry

**Affiliations:** 1 Department of Pathology, University of Washington, Seattle, Washington, United States of America; 2 Center for Cardiovascular Biology, University of Washington, Seattle, Washington, United States of America; 3 Institute for Stem Cell and Regenerative Medicine, University of Washington, Seattle, Washington, United States of America; 4 Department of Bioengineering, University of Washington, Seattle, Washington, United States of America; Katholieke Universiteit Leuven, Belgium

## Abstract

**Background:**

Wnt/β-catenin signaling is an important regulator of differentiation and morphogenesis that can also control stem cell fates. Our group has developed an efficient protocol to generate cardiomyocytes from human embryonic stem (ES) cells via induction with activin A and BMP4.

**Methodology/Principal Findings:**

We tested the hypothesis that Wnt/β-catenin signals control both early mesoderm induction and later cardiac differentiation in this system. Addition of exogenous Wnt3a at the time of induction enhanced cardiac differentiation, while early inhibition of endogenous Wnt/β-catenin signaling with Dkk1 inhibited cardiac differentiation, as indicated by quantitative RT-PCR analysis for β*-myosin heavy chain* (β*-MHC*), *cardiac troponin T (cTnT)*, *Nkx2.5*, and flow cytometry analysis for sarcomeric myosin heavy chain (sMHC). Conversely, late antagonism of endogenously produced Wnts enhanced cardiogenesis, indicating a biphasic role for the pathway in human cardiac differentiation. Using quantitative RT-PCR, we show that canonical Wnt ligand expression is induced by activin A/BMP4 treatment, and the extent of early Wnt ligand expression can predict the subsequent efficiency of cardiogenesis. Measurement of *Brachyury* expression showed that addition of Wnt3a enhances mesoderm induction, whereas blockade of endogenously produced Wnts markedly inhibits mesoderm formation. Finally, we show that Wnt/β-catenin signaling is required for Smad1 activation by BMP4.

**Conclusions/Significance:**

Our data indicate that induction of mesoderm and subsequent cardiac differentiation from human ES cells requires fine-tuned cross talk between activin A/BMP4 and Wnt/β-catenin pathways. Controlling these pathways permits efficient generation of cardiomyocytes for basic studies or cardiac repair applications.

## Introduction

Heart failure is a leading cause of death among all patient populations, in large part due to the heart's limited ability for self-repair. Thus, a cell-based regenerative strategy for cardiac repair would be highly attractive. A variety of cell sources have been identified as candidates for myocardial repair, including skeletal myoblasts [1], various bone marrow stem cells [2], resident cardiac progenitors [3], and pluripotent cells such as embryonic stem (ES) cells or induced pluripotent stem (iPS) cells [4]. Human ES cells are capable of differentiation into definitive cardiomyocytes, as indicated by appropriate contractile function, action potentials and electromechanical coupling (the cells beat synchronously in culture), as well as ultrastructural morphology and gene expression [5], [6], [7].

Significant progress has recently been made toward increasing the efficiency of human ES cell differentiation into cardiomyocytes by harnessing pathways learned from developmental biology. Our group has demonstrated that sequential treatment with activin A and BMP4 results in enhanced generation of mesoderm followed by induction toward a cardiac fate, with 10–50% of cells differentiating into definitive cardiomyocytes [8], [9]. However, before these cells can be used for therapy in humans, it will be necessary to understand the signaling pathways that affect the differentiation and maturation of ES cells and their progeny. Thus, we set out to determine the role of Wnt/β-catenin signaling in human ES cells undergoing cardiac directed differentiation with activin A/BMP4.

The precise role of Wnt pathways in cardiovascular differentiation remains unclear. Early studies in chick and frog embryos showed that canonical Wnt antagonists, crescent and Dikkopf (Dkk), induce cardiac gene expression while Wnt/β-catenin signaling inhibits cardiac differentiation [10], [11]. However, Wnt/β-catenin signaling was shown to enhance cardiac differentiation in pluripotent mouse P19CL6 cells [12]. Recent work from our group and others has partially resolved this discrepancy by showing that canonical Wnt/β-catenin signaling has a biphasic effect on cardiogenesis [13], [14], [15]. Thus, the role of canonical Wnt signaling pathways in cardiogenesis is potent, complex and highly context-dependent.

We hypothesized that Wnt/β-catenin signaling would be an important modulator of cardiac differentiation in human ES cells. Thus, we examined the role of canonical Wnt signaling in the context of activin A/BMP4 cardiac directed differentiation. We found that the expression of several canonical Wnt ligands was induced by activin A/BMP4 during the early stages of differentiation. Prior to mesoderm specification, addition of exogenous canonical Wnt ligand enhanced cardiac differentiation while inhibition of endogenous canonical Wnt signaling reduced the efficiency of cardiac differentiation. Furthermore, inhibition of canonical Wnt signaling at later stages of differentiation enhanced cardiogenesis. Finally, we demonstrated cross-talk between Wnt and BMP pathways at the level of Smad1 phosphorylation. Thus, interplay between Wnt and TGFβ family signaling controls human ES cell differentiation to cardiomyocytes and can be exploited for efficient directed differentiation.

## Results

### Wnt/β-catenin Signaling Regulates Cardiac Differentiation

**Figure 1:**
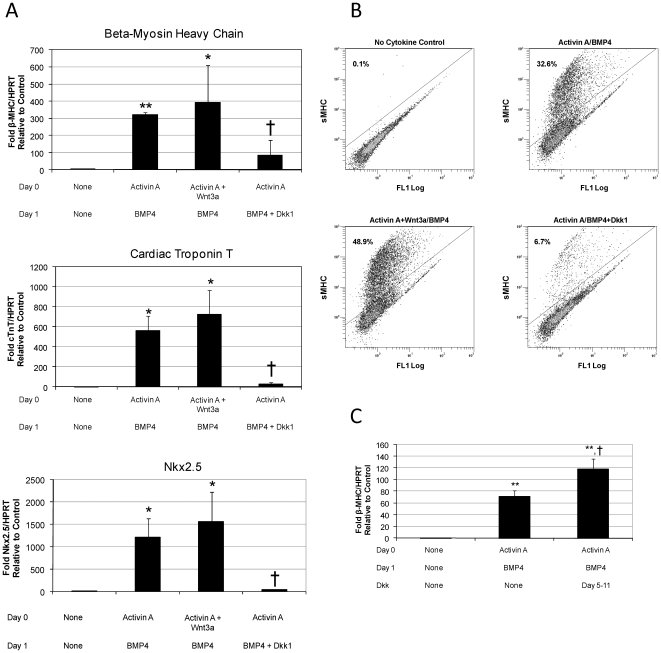
Early Wnt/β-catenin signaling enhances cardiac differentiation. (**A**) Quantitative RT-PCR on day 14 of differentiation shows induction of *β-MHC, cTnT and Nkx2.5* expression by activin A/BMP4 is attenuated with Dkk1. Data are presented as mean fold expression normalized to *HPRT* relative to control cells. (**B**) Flow cytometry analysis for sarcomeric myosin heavy chain (sMHC) on day 21 of differentiation shows enhanced cardiac differentiation with addition of exogenous Wnt3a and decreased cardiac differentiation with Dkk1. (**C**) Late addition of Dkk1 enhanced day 14 *β-MHC* expression relative to samples treated with activin A/BMP4 only. Data are presented as mean fold expression normalized to *HPRT* relative to control cells. *p<0.05 relative to control, **p<0.01 relative to control, †p<0.05 relative to activin A/BMP4, ‡p<0.01 relative to activin A/BMP4.

In an effort to improve the efficiency of cardiac differentiation from human ES cells, our group recently developed a directed differentiation protocol that involves sequential treatment with activin A and BMP4 [8]. This combination of factors results in cultures that typically contain 10–50% cardiomyocytes and spontaneously contract. To examine the role of Wnt/β-catenin signaling in this system, we treated human ES cells undergoing directed differentiation with Wnt3a, a canonical Wnt ligand, or Dkk1, a canonical Wnt inhibitor. Using quantitative RT-PCR, we measured expression of cardiac-specific genes, including *β-myosin heavy chain* (*β-MHC*, aka *MYH7*), a contractile protein, *cardiac troponin T (cTnT)*, a regulator of cardiac muscle contraction, and *Nkx2.5*, a cardiac transcription factor, on day 14 of differentiation. Using the standard activin A/BMP4 directed differentiation protocol, we found over a 300-fold increase in *β-MHC* relative to cultures treated with medium alone without cytokines (p<0.01) (Figure 1A). Addition of Wnt3a enhanced this effect (about 400-fold induction over controls) while addition of Dkk1 drastically reduced cardiac differentiation (only 85-fold induction over controls). Similar results were seen for *cTnT* and *Nkx2.5*. In addition, we used flow cytometry to quantify the proportion of cells expressing sarcomeric myosin heavy chain (sMHC) in six independent experiments. By 21 days of differentiation, 33% of activin A + BMP4 treated cultures were sMHC positive. Of note, we have verified the absence of skeletal muscle differentiation, the only other cell type to express this epitope. Exogenous Wnt3a increased the proportion of sMHC positive cells to 49% while inhibition of canonical Wnt signaling with Dkk1 reduced the population to 7% (Figure 1B). While there was variation in the exact percentage of sMHC expressing cells between experiments, the addition of Wnt3a or Dkk1 consistently enhanced or inhibited cardiac differentiation relative to standard activin A/BMP4 treatment, respectively. The marked inhibition of cardiac differentiation by Dkk1 indicates that endogenously produced Wnt signals are required in the early stages of differentiation induced by exogenous activin A and BMP4.

To determine if other mesodermal lineages emerge using activin A/BMP4 directed differentiation, we performed quantitative RT-PCR for *SM22*α and *VE-cadherin*, markers of smooth muscle and endothelium, respectively (Figure S1). Using the standard activin A/BMP4 treatment, we found only about a 3-fold increase in *SM22*α and a 5-fold increase in *VE-cadherin*. Enhancing canonical Wnt signaling using exogenous Wnt3a or blocking endogenous canonical Wnt signaling with Dkk1 had no significant effect on expression of either of these markers. Taken together, these data suggest that our directed differentiation system highly enriches the cardiomyocyte yield, and manipulation of Wnt/β-catenin signaling in this context does not alter differentiation of smooth muscle or endothelial lineages.

### Effects of Wnt/β-catenin Signaling on Cardiac Differentiation are Stage-Specific

We have previously shown that inhibition of Wnt/β-catenin signaling after mesoderm specification can enhance cardiac differentiation in mouse ES cells [13]. Thus, we hypothesized that human ES cells would also show increased cardiac differentiation in response to inhibition of Wnt/β-catenin signaling during later stages of differentiation. Dkk1 was added to human ES cells undergoing directed differentiation for 4–10 days following BMP4 treatment (days 5–11 of differentiation). Quantitative RT-PCR showed a 70% increase in *β-MHC* expression in cultures treated with activin A/BMP4 plus late Dkk1 compared to cultures treated with activin A/BMP4 only (Figure 1C). Thus, inhibition of canonical Wnt signaling can enhance cardiac differentiation in human ES cells when Dkk1 is added later in differentiation, after definitive mesoderm has formed.

### Canonical Wnts are Expressed During Early activin A/BMP4-Mediated Cardiac Differentiation

**Figure 2:**
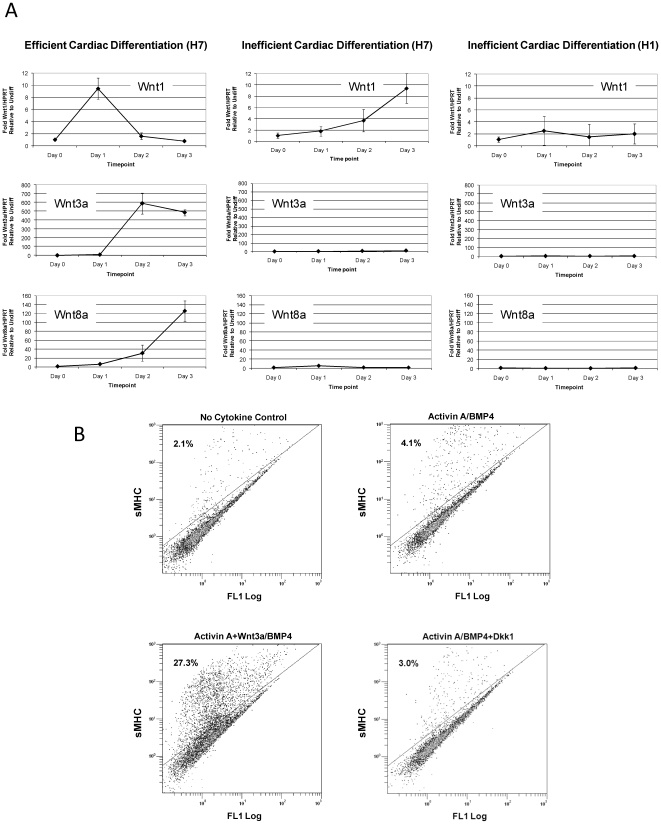
Canonical Wnt expression during mesoderm specification correlates with the efficiency of cardiac differentiation. Quantitative RT-PCR for canonical Wnt ligands *Wnt1*, *Wnt3a* and *Wnt8a*. Data are presented as mean fold expression normalized to *HPRT* relative to undifferentiated cells. (**A**) Representative of cultures of H7 hES cells that showed efficient and inefficient directed differentiation to cardiomyocytes (>25% and <5% sMHC+, respectively), as well as H1 hES cells that are poorly cardiogenic. (**B**) Cultures that routinely generated <5% cardiomyocytes were treated with Wnt3a or Dkk1 in addition to activin A and BMP4. After 17 days of differentiation following activin A treatment, cells were stained for sMHC. Exogenous Wnt3a partially rescued inefficient cardiac differentiation.

The observation that Dkk1 inhibits cardiac differentiation when added at day 1 points to the presence of endogenous canonical Wnt activity at this stage of differentiation. Thus, we next studied expression of canonical Wnt ligands during early directed differentiation of H7 human ES cells using quantitative RT-PCR. Key canonical Wnts, including *Wnt1*, *Wnt3a*, and *Wnt8a* were up-regulated during this time (Figure 2A). *Wnt1* was induced 8–10 fold on day 1 and decreased thereafter. *Wnt3a* was induced ∼600-fold on day 2 and remained high on day 3. *Wnt8a* was induced 30-fold on day 2 and 120-fold on day 3. These studies confirm the induction of endogenous canonical Wnt ligands during cardiac differentiation directed by activin A and BMP4, and reveal that the various canonical Wnt ligands are differentially upregulated.

We have observed that the activin A/BMP4 directed differentiation protocol is not always successful. For reasons that are not yet clear, some runs yield less than 5–10% cardiomyocytes. Interestingly, a different pattern of early canonical Wnt expression was observed in cultures of H7 human ES cells that ultimately yielded low numbers of cardiomyocytes (Figure 2A). The most dramatic differences in expression levels were observed for *Wnt3a* and *Wnt8a*. *Wnt3a* expression peaked at 11.6 fold at day 3 of differentiation in cultures containing low cardiac yield. This is in stark contrast to the ∼600-fold peak observed in cultures with high cardiomyocyte yields. *Wnt8a* expression increased to 5.1 fold by day 1 and then dropped back down to baseline levels, in contrast to the 120-fold induction in high yield runs. We also examined canonical Wnt ligand expression during differentiation of H1 human ES cells, which are less cardiogenic than H7 cells, and generate cultures that are <1% cardiomyocytes in our hands (data not shown). In H1 cells, *Wnt1, Wnt3a*, and *Wnt8a* were not significantly upregulated during mesoderm specification following treatment with activin A/BMP4. This suggests that the pattern of canonical Wnt expression during early differentiation correlates with the efficiency of cardiogenesis.

To assess the effect of modulating Wnt/β-catenin signaling in cultures that differentiate poorly, we administered exogenous Wnt3a or Dkk1 during the early stages of differentiation, as in the previously described experiments. Interestingly, we found that addition of Wnt3a can rescue cultures of H7 human ES cells that demonstrated inefficient cardiac differentiation in response to activin A/BMP4 (Figure 2B). Cultures treated with activin A/BMP4 were only 4% sMHC+, whereas the early addition of exogenous Wnt3a increased the cardiomyocytes population to 27%. Inhibition of Wnt/β-catenin signaling with Dkk1 resulted in cultures that were 3% sMHC+. This effect is less dramatic than what was observed in the previously mentioned experiments, likely due to the decrease in endogenous canonical Wnt signaling in these cultures. Taken together, these studies show that Wnt/β-catenin signaling is necessary for cardiac differentiation in human ES cells and absence of endogenously produced Wnts correlates with poor cardiac differentiation.

### Wnt/β-catenin Signaling Regulates Mesoderm Specification

**Figure 3:**
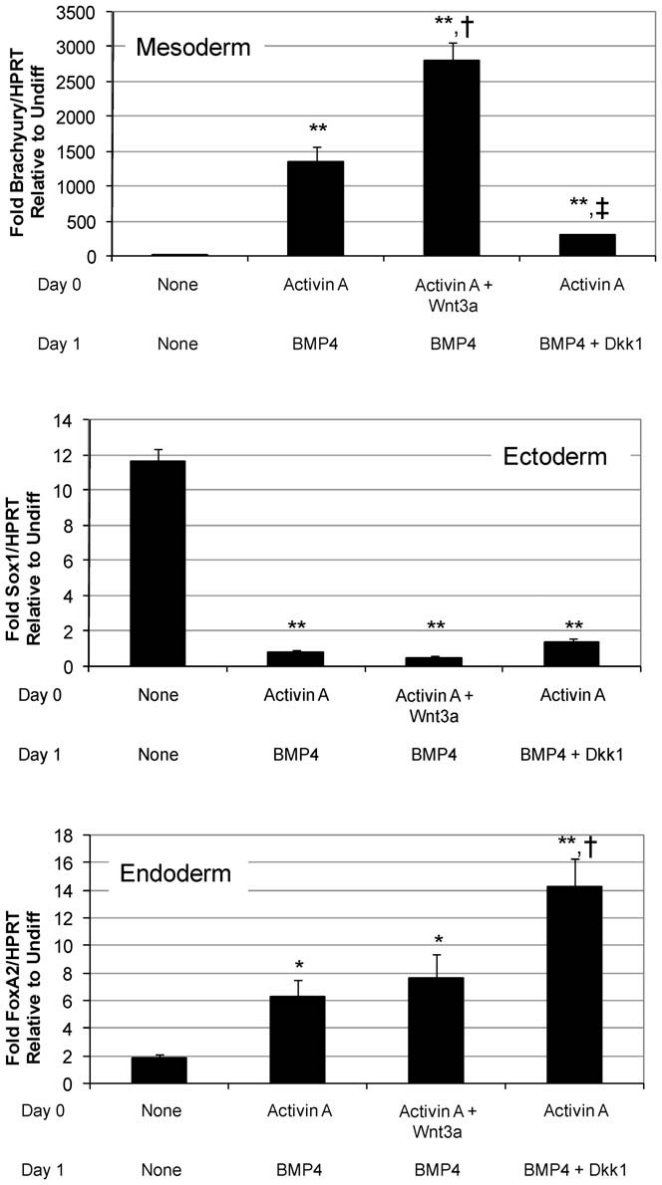
Wnt/β-catenin signaling enhances mesoderm induction. Quantitative RT-PCR analysis on day 2 of differentiation shows induction of *Brachyury* (mesoderm), *FoxA2* (endoderm), and *Sox1* (ectoderm). *Brachyury* expression induced by activin A/BMP4 was enhanced by Wnt3a and reduced by Dkk1. Data are presented as mean fold expression normalized to *HPRT* relative to undifferentiated cells + SEM. *p<0.05 relative to control, **p<0.01 relative to control, †p<0.05 relative to activin A/BMP4, ‡p<0.01 relative to activin A/BMP4.

As a first step to elucidate the mechanism by which early Wnt/β-catenin signals regulate cardiogenesis in human ES cells, we collected RNA at early time points to measure the induction of germ-layer specific markers, namely *Brachyury T* (mesoderm), *Sox1* (ectoderm), and *FoxA2* (endoderm). We have previously shown that in our directed differentiation system, *Brachyury* expression peaks around two days after addition of activin A [9]. Thus, we used quantitative RT-PCR to measure *Brachyury* expression on day 2 of differentiation in cells treated with activin A/BMP4 (standard directed differentiation) compared with cells treated with activin A/BMP4 plus exogenous Wnt3a or Dkk1 to block endogenous canonical Wnt signaling. Samples collected on day 0, prior to induction with activin A, were used to determine baseline expression. While addition of Wnt3a enhanced *Brachyury* expression, addition of Dkk1 greatly attenuated mesoderm induction normally seen with our directed differentiation protocol (Figure 3). These data suggest that endogenous canonical Wnts are necessary for mesoderm formation and subsequent cardiogenesis in human ES cells. Cells treated with control medium showed the highest level of *Sox1* expression, consistent with the observation that ES cells spontaneously differentiate into ectoderm in the absence of factors mimicking the primitive streak environment. Although exogenous Wnt3a had no effect on *FoxA2* expression, Wnt antagonism with Dkk1 enhanced expression of this endoderm marker.

To investigate the effects of canonical Wnt signaling on cell death and proliferation during this stage of differentiation, we performed cell counts, annexin V staining for apoptotic cells, and propidium iodide staining for cell cycle analysis (Figure S2). Cultures treated with activin A/BMP4 had roughly 40% fewer adherent cells than non-treated controls on day 2 of differentiation in association with an increase in non-adherent cells. Wnt3a or Dkk1 treatment did not significantly alter cell number. Among the adherent cell population, roughly 15% of the cells were annexin V positive in the control and activin A/BMP4 treated groups. This increased to 20% with Wnt3a addition and 30% with Dkk1. In addition, there were no significant changes in cell proliferation between treatment groups, with all cultures showing roughly 50% of cells in G1 phase, 25–30% in S phase, and 20% in G2/M phase. Overall, these effects are mild compared to the drastic effects on *Brachyury* and cardiac gene expression, suggesting that the primary role of Wnt/β-catenin signaling in this context is in driving differentiation rather than influencing cell death or proliferation. Interestingly, cell fractionation showed no increase in nuclear localization of β-catenin in undifferentiated (day 0) vs. differentiated (day 2) cultures (Figure S3), suggesting regulation distal to nuclear translocation.

### Canonical Wnt Signaling Regulates Smad Activation

**Figure 4:**
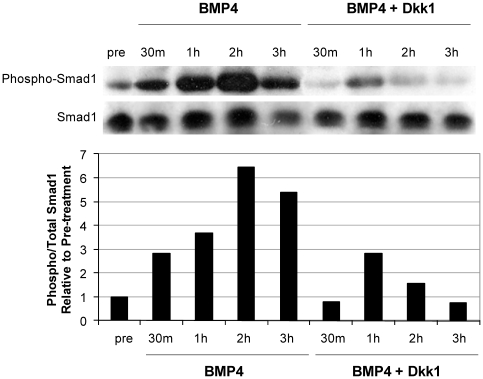
Wnt/β-catenin signaling regulates Smad1 activation. High-density undifferentiated human ES cells were treated with activin A for 24 hours followed by BMP4 or BMP4 plus Dkk1. Lane marked as “pre” indicates cells collected prior to cytokine treatment. Western blot shows probing for C-terminal phosphorylated Smad1 (p-Smad1) and total Smad1 at 30 m, 1 h, 2 h and 3 h following BMP4. Densitometry data are presented as phosphorylated Smad1 normalized to total Smad1 relative to pretreated cultures. Treatment with BMP4 increased phosphorylation of Smad1, which was reduced by inhibition of canonical Wnt signaling with Dkk1.

We next explored intracellular signaling pathways to determine how activin A/BMP4 signaling synergizes with canonical Wnt signaling to drive mesoderm and ultimately cardiac differentiation. Using Western blotting, we monitored Smad1 or Smad2 activation by probing for phosphorylated Smads. We found that Dkk1 reduced activation of Smad1 in response to BMP4 stimulation (Figure 4), suggesting that endogenous canonical Wnt signaling enhances phospho-Smad1 production or stability in the context of the activin A/BMP4 cardiac directed differentiation system.

## Discussion

Wnt signaling plays a key role in the differentiation and morphogenesis of many cell types and tissues. However, the effects of Wnt/β-catenin signaling on cardiac differentiation have been difficult to pinpoint due to conflicting data. While studies in frog and chick embryos suggested that Wnt/β-catenin signaling suppressed cardiac differentiation, evidence from mouse P19CL6 teratocarcinoma cells indicated a positive role for Wnt/β-catenin signaling in cardiogenesis [10], [11], [12]. Our group and others addressed these discrepancies by showing that Wnt/β-catenin signaling has a biphasic effect, whereby cardiac differentiation is enhanced prior to gastrulation and inhibited after gastrulation [13], [14], [15]. By using transgenic zebrafish embryos, we showed that Wnt/β-catenin signaling prior to gastrulation is necessary for mesoderm induction but rapidly switches to an anti-cardiogenic effect after gastrulation [13]. Similarly, in mouse ES cells, early activation of Wnt/β-catenin signaling enhances mesoderm and cardiac induction, as indicated by increased *Brachyury T* and α*-myosin heavy chain* (α*-MHC*) expression, respectively. However, activation of the same pathway inhibits cardiac differentiation at later timepoints, likely by directing cells towards alternative mesodermal fates [13]. Interestingly, it was recently shown that in mouse ES cells, Dkk1 expression is activated by MesP1, suggesting that this transcription factor, expressed in pre-cardiac mesoderm, may promote cardiac differentiation by inhibiting Wnt/β-catenin signals [16].

In the present study, we showed that Wnt/β-catenin signaling modulates activin A/BMP4-mediated cardiac differentiation of human ES cells. During early stages of differentiation, addition of exogenous Wnt3a enhanced cardiac differentiation while inhibition of canonical Wnt signaling with Dkk1 decreased cardiogenesis. These data indicate that the ability of activin A/BMP4 to induce cardiac differentiation depends on endogenous Wnt signaling. Indeed, induction of multiple canonical Wnt ligands can be observed during early directed differentiation, which leads to high cardiomyocyte yields. On the other hand, addition of Dkk1 at later stages of differentiation increased cardiogenesis, showing that Wnt/β-catenin signaling has a biphasic effect in human ES cells, similar to our previous findings in zebrafish embryos and mouse ES cells [13]. Thus, these studies show that Wnt/β-catenin and activin A/BMP4 signaling interact to regulate cardiac differentiation in human ES cells.

Recent work from other groups shows that activin/BMP signaling, along with canonical Wnts, modulates primitive streak and subsequent germ layer differentiation in mouse and human ES cells [17], [18], [19]. Our data support these previous findings, showing that activin A/BMP4 induction of the mesoderm-specific transcription factor *Brachyury* is enhanced by addition of exogenous Wnt3a and repressed by addition of Dkk1. The data presented here suggest that canonical Wnt signaling is a key regulator of mesoderm specification and subsequent cardiogenesis in human ES cells undergoing directed differentiation with activin A/BMP4. Interestingly, the pattern of canonical Wnt ligand expression during the first three days of differentiation was drastically different in H7 cultures that ultimately showed >25% cardiomyocytes compared to H7 and H1 cultures that generate only 5–10% or <1% cardiomyocytes, respectively. Furthermore, addition of exogenous Wnt3a was able to rescue inefficient cardiac directed differentiation in some cases, increasing the percent sMHC+ cells by more than 6-fold (Figure 2). These data indicate that variations in endogenous signaling pathways could account for much of the variation in efficiency encountered with this directed differentiation protocol.

Our data also show that Wnt/β-catenin signaling modulates signaling events downstream of BMP4. Activin A and BMP4 are members of the TGF-β superfamily. Activin A signals through C-terminal phosphorylation and subsequent activation of Smad2 while BMP4 signaling results in similar activation of Smad1. These phosphorylated Smads interact with Smad4 and enter the nucleus to serve as transcription factors. It has been suggested that canonical Wnt signaling stabilizes Smads by inhibiting GSK3, which has been shown to phosphorylate Smad1 at a site, distinct from that phosphorylated by the BMP receptor, which targets it for ubiquitination and subsequent degradation [20]. Indeed, we show that the ability of BMP4 to induce receptor-mediated phosphorylation of Smad1 is dramatically reduced in the presence of Dkk1. In contrast, Wnt3a did not enhance Smad2 activation following activin A treatment (data not shown). Thus, the cross-talk between canonical Wnt and TGF-β signaling pathways likely involves direct interactions between downstream signaling effectors in addition to co-regulation of target genes.

We have shown the importance of activin A, BMP4 and Wnt/β-catenin signaling in driving cardiac differentiation in human ES cells. As in development, many other signaling pathways are likely modulating cardiogenesis in this system. It will be essential to address the interactions between these various pathways to maximize cardiac differentiation from human ES cells. This will greatly improve our understanding of human cardiac development and enhance our ability to produce cardiomyocytes for future basic studies and cardiac repair applications.

## Materials and Methods

### Maintenance of Human Embryonic Stem Cells

H7 and H1 human ES cells were maintained as previously described [21]. In brief, cells were grown on Matrigel-coated plates in mouse embryonic fibroblast-conditioned medium (MEF-CM) supplemented with basic fibroblast growth factor (bFGF) at a concentration of 8 ng/mL. Cells were passaged every 4–6 days using collagenase IV (Gibco) to detach the cells from the Matrigel-coated plates.

### Cardiac Directed Differentiation

For cardiac directed differentiation, undifferentiated human ES cells were replated and allowed to grow to confluence as high-density monolayers on 24-well plates. Cells were then treated with 100 ng/mL recombinant activin A in RPMI medium (Invitrogen) supplemented with B27 (Invitrogen) for 24 hours followed by 10 ng/mL human recombinant BMP4 for 4 days. RPMI-B27 medium was replaced every 2 days thereafter. When applicable, 100 ng/mL recombinant Wnt3a was added with activin A or 200 ng/mL recombinant Dkk1 was added with BMP4. Dkk1 lot activity was verified using HEK cells expressing luciferase driven by a beta-catenin activated reporter (BAR) [22]. Recombinant mouse Wnt3a was purchased from Millipore. Recombinant mouse activin A, human BMP4 and human Dkk1 were purchased from R+D Systems.

### RT-PCR

Total RNA was isolated using the RNeasy Miniprep kit (Qiagen). First-strand cDNA was synthesized from 1 µg total RNA using the Superscript II enzyme kit (Invitrogen) and random hexamer primers. Quantitative PCR was performed using SYBR Green PCR kit on a 7900HT Fast-Real-Time PCR System (Applied Biosystems). The copy number for each transcript is expressed relative to that of *HPRT*, which is used as an internal control, and normalized to a value of 1.0 for undifferentiated cells or control cells not treated with exogenous cytokines.

### Flow Cytometry

For analysis of cardiac differentiation, cell cultures were harvested with TrypLE (Invitrogen) plus 100 U/mL DNase (Invitrogen) and fixed in cold methanol. Cells were incubated with purified anti-sarcomeric myosin heavy chain primary antibody (MF20, Developmental Studies Hybridoma Bank, 1∶100) overnight at 4°C followed by PE-conjugated goat anti-mouse secondary antibody (Jackson, 1∶150) for one hour at room temperature. Analysis was performed using an FC500 machine (BD).

For apoptosis analysis, dissociated cells were stained with annexin V-APC antibody (eBioscience) according to the manufacturer's instructions. Briefly, cells were incubated in 100 uL annexin binding buffer with 5 uL annexin V-APC antibody for 15 minutes at room temperature, washed, and analyzed.

Cell cycle analysis was performed by fixing cells in cold ethanol followed by incubation with propidium iodide (40 ug/mL, Molecular Probes) and RNase (100 ug/mL, Macherey-Nagel) for one hour at 37°C. FlowJo software was used to determine the percent of cells in G1, S, or G2/M phases.

### Western Blotting

Cells were lysed with sample buffer containing Tris-HCl (50 mM), SDS (1%), glycerol (10%), complete protease inhibitor (Roche), and phosphatase inhibitors (sodium fluoride, sodium pyrophosphate, sodium orthovanadate, 1 mM each). Samples were sheared using a 20 gauge needle. For samples requiring separation of cytoplasmic and nuclear proteins, we utilized the NE-PER kit (Pierce) according to the manufacturer's instructions. Protein concentration was determined using a bicinchoninic acid assay (BCA) (Pierce). Samples were boiled, and 10 µg total protein was loaded into 10% acrylamide gels. Protein was transferred to PVDF membranes. Blot were blocked in 0.5% milk in Tris-buffered saline plus 0.1% Tween, incubated in primary antibody for 10 minutes, rinse with TBS-Tween three times, incubated in secondary antibody for 10 minutes, and rinsed with TBS-Tween three using a SNAP id apparatus (Millipore). Visualization was accomplished using chemiluminescence (Pierce). Primary antibodies used were: anti-Smad1 (Cell Signaling, 1∶1000), anti-phosphoSmad1 (Cell Signaling, 1∶1000), anti-β-catenin (Abcam, 1∶1000), anti-β-actin (Abcam, 1∶1500), anti-β-tubulin (Cell Signaling, 1∶250). Secondary antibodies used were HRP-conjugated goat anti-rabbit or goat anti-mouse (Jackson, 1∶5000). Densitometry analysis was performed using Image J software.

## Supporting Information

Figure S1 Early Wnt/β-catenin signaling does not affect other cardiovascular lineages during cardiac directed differentiation. Quantitative RT-PCR on day 14 of differentiation shows a small induction of SM22α and VE-cadherin by activin A/BMP4 treatment. These effects are not significantly altered by treatment with either Wnt3a or Dkk1.
(0.67 MB TIF)

Figure S2 Early Wnt/β-catenin signaling has a modest effect on apoptosis and proliferation. (A) Shown are both adherent and non-adherent cell counts from 3 wells of a 24-well plate for each treatment group. (B) Samples were analyzed by flow cytometry for annexin V to identify apoptotic cells. (C) Samples were incubated with propidium iodide and analyzed by flow cytometry for DNA content.
(0.75 MB TIF)

Figure S3 β-catenin is present in the nuclei of undifferentiated ES cells and early differentiated cells. Shown are Western blots of cytoplasmic and nuclear fractions from day 0 (undifferentiated) and day 2 (mesoderm) cells. Probing for β-catenin shows robust expression in both the cytoplasmic and nuclear fractions at both timepoints. Also shown is β-actin as a loading control and β-tubulin to verify minimal contamination of cytoplasmic contents in the nuclear fractions. C = cytoplasmic fraction, N = nuclear fraction.
(0.11 MB TIF)
