# Supplementary figures and images for: Endogenous Wnt/β-Catenin Signaling Is Required for Cardiac Differentiation in Human Embryonic Stem Cells
Source: PLoS One. 2010 Jun 15;5(6):e11134. doi: 10.1371/journal.pone.0011134 (PMC2886114; doi:10.1371/journal.pone.0011134)

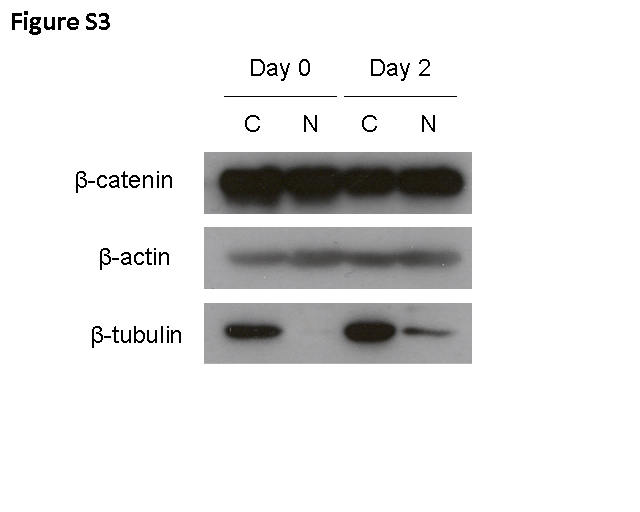

Supplement: Figure S3 — β-catenin is present in the nuclei of undifferentiated ES cells and early differentiated cells. Shown are Western blots of cytoplasmic and nuclear fractions from day 0 (undifferentiated) and day 2 (mesoderm) cells. Probing for β-catenin shows robust expression in both the cytoplasmic and nuclear fractions at both timepoints. Also shown is β-actin as a loading control and β-tubulin to verify minimal contamination of cytoplasmic contents in the nuclear fractions. C = cytoplasmic fraction, N = nuclear fraction. (0.11 MB TIF) [file pone.0011134.s003.tif]
